# Efficacy of Immune Checkpoint Inhibitors against Advanced or Metastatic Neuroendocrine Neoplasms: A Systematic Review and Meta-Analysis

**DOI:** 10.3390/cancers14030794

**Published:** 2022-02-04

**Authors:** Eun-Joo Park, Hyo-Jung Park, Kyung-Won Kim, Chong-Hyun Suh, Changhoon Yoo, Young-Kwang Chae, Sree Harsha Tirumani, Nikhil H. Ramaiya

**Affiliations:** 1 Asan Medical Center, Department of Radiology and Research Institute of Radiology, University of Ulsan College of Medicine, Seoul 05505, Korea; yoursxion@naver.com (E.-J.P.); kyungwon_kim@amc.seoul.kr (K.-W.K.); chonghyunsuh@amc.seoul.kr (C.-H.S.); 2 Asan Medical Center, Department of Oncology, University of Ulsan College of Medicine, Seoul 05505, Korea; yooc@amc.seoul.kr; 3 Feinberg School of Medicine, Robert H. Lurie Comprehensive Cancer Center, Department of Medicine, Northwestern University, Chicago, IL 60611, USA; young.chae@northwestern.edu; 4 University Hospitals Cleveland Medical Center, Department of Radiology, Case Western Reserve University, 11100 Euclid Ave., Cleveland, OH 44106, USA; SreeHarsha.Tirumani@uhhospitals.org (S.H.T.); Nikhil.Ramaiya@uhhospitals.org (N.H.R.)

**Keywords:** checkpoint inhibitors, immunotherapy, neuroendocrine neoplasm, treatment efficacy

## Abstract

**Simple Summary:**

Neuroendocrine neoplasms (NENs) are relatively rare neoplasms, but their incidence is rising. Despite the recent understanding of and advances in the treatment of NENs, the clinical outcomes of patients with NENs, especially high-grade NENs, are poor. This meta-analysis was conducted to systematically evaluate the effectiveness of immune checkpoint inhibitors (ICIs) in patients with advanced or metastatic NENs. In 10 studies with 464 patients, the pooled overall response rate was 15.5% (95% confidence interval, 9.5–24.3%), but the response differed according to the primary tumor site, tumor differentiation, and drug regimen. Poorly differentiated NENs consistently showed a better overall response rate than well-differentiated NENs in multiple sensitivity analyses, suggesting that different expression of immune checkpoints and tumor mutational burden may influence the treatment efficacy of ICIs for advanced or metastatic NENs. The variation in treatment efficacy of ICIs according to tumor differentiation and drug regimen should be considered for patient-tailored management.

**Abstract:**

We performed a systematic review and meta-analysis of the treatment efficacy of immune checkpoint inhibitors (ICIs) in advanced/metastatic neuroendocrine neoplasms (NENs). MEDLINE and EMBASE were searched to identify studies that provide data on treatment response and/or survival outcomes of advanced/metastatic NEN patients treated with ICIs. The overall response rate (ORR) was pooled using a random-effects model. Meta-regression was performed to explore factors influencing the ORR. Individual patient data (IPD) meta-analysis of survival was performed using stratified Cox regression. Ten studies (464 patients) were included. The overall pooled ORR was 15.5% (95% confidence interval (CI), 9.5–24.3%), and it varied according to the primary site (thoracic, 24.7%; gastro–entero–pancreatic, 9.5%), tumor differentiation (poorly differentiated, 22.7%; well-differentiated, 10.4%), and drug regimen (combination, 25.3%; monotherapy, 10.1%). All these variables significantly influenced the ORR. Tumor differentiation was associated with both overall survival and progression-free survival (hazard ratio of poorly differentiated tumors, 4.2 (95% CI, 2.0–8.7) and 2.6 (95% CI, 1.6–4.4), respectively). Thus, the treatment efficacy of ICIs for advanced/metastatic NENs varied according to primary site, tumor differentiation, and drug regimen. Poorly differentiated NENs showed a better ORR than well-differentiated NENs but had a negative impact on survival.

## 1. Introduction

Neuroendocrine neoplasms (NENs) represent a heterogeneous group of tumors originating from the neuroendocrine cells of various organs [1]. The behavior and prognosis of NENs are highly variable and depend on the site of origin, tumor grade, and differentiation [2]. Although NENs are relatively uncommon tumors, recent reports have shown that their incidence and prevalence are growing worldwide [3,4,5]. For patients with advanced/metastatic NENs, systemic therapy is the only available treatment option. The recommended regimens include sunitinib, everolimus, somatostatin analogue, peptide receptor radionuclide therapy, and cytotoxic chemotherapy [6,7,8]. Despite recent progress in the understanding of the pathophysiology and molecular biology, as well as advances in treatment strategies for NENs [9,10,11], the clinical outcomes for individuals with high-grade NENs are poor, with a reported median overall survival (OS) of 11 months and median progression-free survival (PFS) of 4 months for patients with poorly differentiated (PD) neuroendocrine carcinoma (NEC) [12,13].

Immune checkpoint inhibitors (ICIs) have made great breakthroughs in cancer therapy and have gained approval for the treatment of various types of cancer [8,14]. In the case of NENs, ICIs were approved for two distinct tumor types: Merkel cell carcinoma and small cell lung cancer [15,16,17,18], both of which have high tumor mutational burden (TMB) and environmental causes of immunogenicity [19]. For other NENs, several early phase trials and clinical studies have evaluated the efficacy of ICIs and given us a preliminary understanding of their role. To date, however, no attempt has been made to generate an evidence-based comprehensive summary of these agents from the scattered individual studies. We thus conducted this systematic review and meta-analysis of the antitumor activity and clinical outcomes of ICI-treated advanced or metastatic NEN patients using currently available published data, which may help further characterize and guide future studies on the role of ICIs in the treatment of NENs.

## 2. Materials and Methods

### 2.1. Search Strategy

The study was performed in accordance with the standard guidelines of Preferred Reporting Items for Systematic Reviews and Meta-Analyses (PRISMA) [20] and PRISMA for Individual Patient Data systematic reviews (PRISMA-IPD) [21]. This study was registered in OSF Registries (osf.io/3tgd4). A comprehensive search of MEDLINE and EMBASE was conducted by two reviewers (E.J.P. and H.J.P.) independently to identify relevant studies published before 1 April 2021. The detailed search strategy is shown in Table S1. The search was limited to human subjects and English-language studies.

### 2.2. Eligibility Criteria

The studies identified from the two databases were combined and duplicate studies were removed from the list. Titles and abstracts were screened for relevance. The relevant full texts were meticulously evaluated for compliance with the inclusion criteria listed below. To expand the search, the bibliographies were also screened for potentially suitable studies. Two reviewers (E.J.P. and H.J.P.) independently selected the studies, and disagreements, if any, were resolved by discussion. Based on the population, intervention, comparison, outcome, study design (PICOS) approach [22], studies fulfilling the following criteria were included: (a) population: patients diagnosed with NENs; (b) intervention: NEN patients treated with ICIs; (c) outcomes: treatment responses or survival endpoints (OS and/or PFS) assessed using Response Evaluation Criteria In Solid Tumors (RECIST) 1.1 [23]; and (d) study design: clinical trials and observational studies (prospective or retrospective). The exclusion criteria were: (a) case reports, reviews, letters, editorials, and conference abstracts; (b) no study of ICI treatment in NEN patients; (c) studies with insufficient response assessment data; (d) studies with cohort overlap. For studies with overlapping cohort, the study with the longest follow-up was selected.

### 2.3. Data Extraction

Two reviewers (E.J.P. and H.J.P.) independently extracted the relevant data from the included studies, and disagreements, if any, were resolved by discussion. The following data were extracted: (a) study characteristics, including authors, year of publication, and study design; (b) demographic and clinical characteristics of the patients, including sample size, types of administered ICI, primary tumor site, and tumor grade and/or differentiation according to the most recent World Health Organization (WHO) classification systems per site [10,24,25,26]; (c) overall response rate (ORR) based on RECIST 1.1; (d) OS and PFS based on RECIST 1.1 [27,28]. The IPD for the OS and PFS was reconstructed by extracting the data from the given Kaplan–Meier curves using a digital software (WebPlotDigitizer v 4.5, https://automeris.io/WebPlotDigitizer, accessed on 12 October 2021) in accordance with the method proposed by Guyot et al. [29]. At all times, we used the endpoint definitions and analyses reported by authors and made no attempts to reclassify or recalculate the values published in the original reports.

### 2.4. Definition of Endpoints for Treatment Efficacy

Endpoints were defined in accordance with guidelines published by the United States Food and Drug Administration [28]. As a response-related endpoint, the ORR, a direct measure of the tumoricidal effects of a treatment, was defined as the proportion of patients showing a complete or partial response per RECIST 1.1. OS and PFS were used as survival endpoints. OS was defined as the time from treatment initiation to death from any cause. PFS was defined as the time from treatment initiation to progressive disease (according to RECIST 1.1) or to death from any cause. Patients were considered censored for a given endpoint if they did not experience this endpoint during their follow-up period.

### 2.5. Quality Assessment

Two reviewers (E.J.P. and H.J.P.) independently reviewed the risk of bias and methodologic quality of the included studies. The Risk of Bias In Non-randomized Studies of Interventions (ROBINS-I) tool [30] was used. Any discrepancy was resolved by discussion.

### 2.6. Statistical Analysis

For pooling the ORR, considering the characteristics of the study patients and the treatment differed between studies, we used the random-effects model with inverse-variance weighting to generate the summary estimate of the magnitude of effect. DerSimonian–Laird random-effect models [31] were constructed to synthesize the pooled ORR with 95% confidence intervals (CIs). The heterogeneity between studies was assessed with I^2^ statistics and a Cochran Q test. An I^2^ > 50% or *p* < 0.1 in the Q test indicated substantial heterogeneity [32]. The publication bias was estimated using funnel plots and Begg’s test [33].

Subgroup analyses were performed to calculate the pooled ORR of each group of primary tumor site (i.e., thoracic [lung or thymus origin] or gastro–entero–pancreatic [GEP]), tumor differentiation (well-differentiated [WD] or PD), and drug regimen (ICI monotherapy or combinations of ICIs). Grade 3 neuroendocrine tumors (NETs) were included in the WD tumor group. Meta-regression analysis was performed to identify influencing factors for the ORR.

With regard to survival outcomes, using the reconstructed IPD, the OS and PFS were calculated using the Kaplan–Meier method. The influence of the primary tumor site, tumor differentiation, and drug regimen on the OS and PFS was assessed using one-stage stratified Cox proportional-hazard regression analysis to account for clustering at the level of each study [34]. Statistical analyses were performed with R, version 4.0.3 (R Foundation for Statistical Computing, Vienna, Austria).

## 3. Results

### 3.1. Study Search and Characteristics

**Figure 1:**
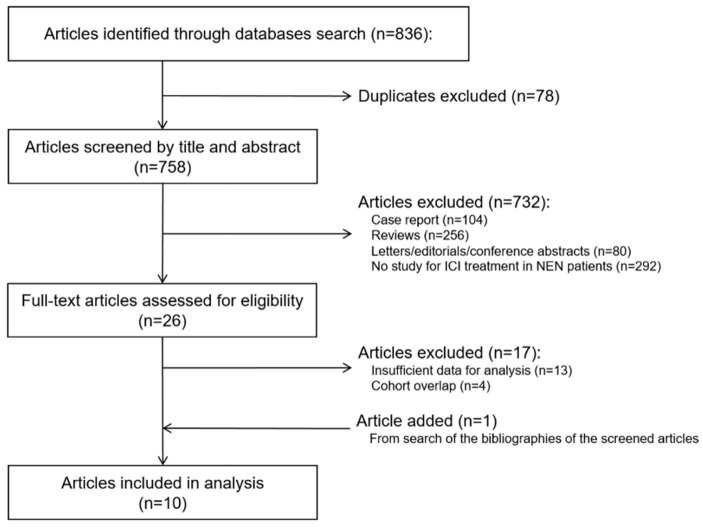
Flow diagram of the study selection. ICI, immune checkpoint inhibitors; NEN, neuroendocrine neoplasm.

The study search process is outlined in Figure 1. The characteristics of the 10 included studies [19,35,36,37,38,39,40,41,42,43], which involved a total of 464 NEN patients, are summarized in Table 1. Seven of these studies were nonrandomized clinical trials (two phase I studies and five phase II studies), and three were retrospective studies. Two studies included only large-cell NECs (LCNECs) of the lung. The remaining eight studies included NENs of various primary sites, including the lung, pancreas, and gastrointestinal tract. All studies included patients with advanced or metastatic NENs, and six studies specified that the patients had progressed during previous systemic treatment(s). Two studies included only PD tumors (large-cell NEC of the lung), two included only WD tumors, and six included both. The ICI regimens varied between studies; six studies involved ICI monotherapy (three with pembrolizumab, one with nivolumab or pembrolizumab, one with toripalimab, and one with spartalizumab) only, two studies reported on an ICI combination therapy (ipilimumab and nivolumab) only, and two studies described both ICI monotherapy and combination therapy.

According to the ROBINS-I tool, the risk of bias for overall risk of bias for the included studies was low in seven studies and moderate in three studies (Figure S1).

### 3.2. Meta-Analysis

#### 3.2.1. Response-Related Endpoint

**Figure 2:**
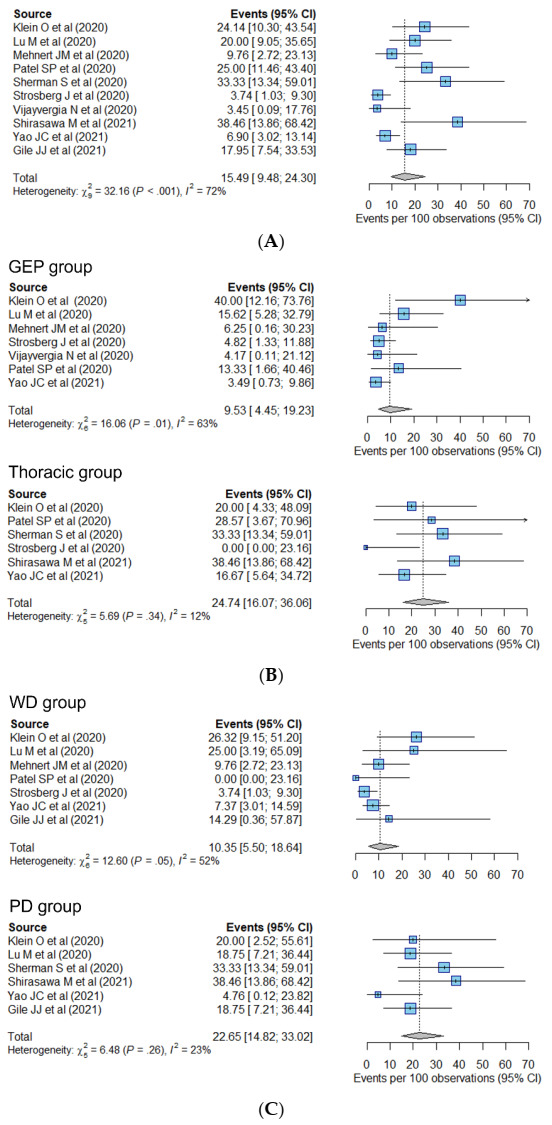

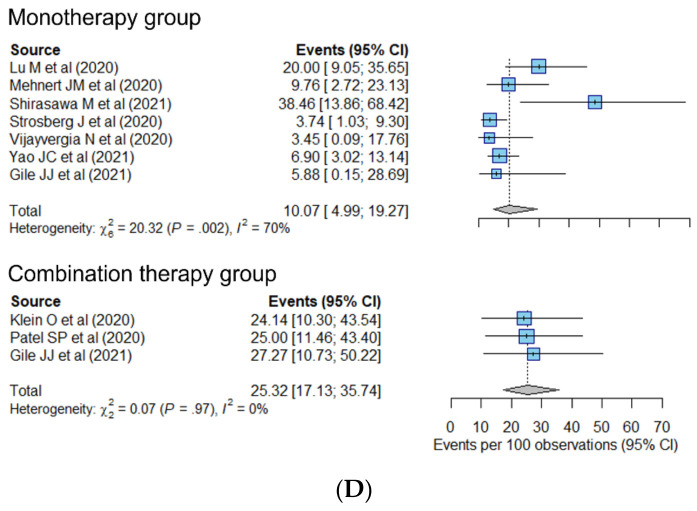
Forest plots showing the pooled estimate of the overall response ratio of (**A**) all studies, and of subgroups stratified by (**B**) the primary site, (**C**) tumor differentiation, and (**D**) drug regimen. The pooled overall response rate was 15.5% for all studies (**A**), 9.5% in the GEP group and 24.7% in the thoracic group (**B**), 10.4% in the WD group and 22.7% in the PD group (**C**), and 10.1% in monotherapy group and 25.3% in combination therapy group (**D**). CI, confidence interval; GEP, gastro–entero–pancreatic; PD, poorly differentiated; WD, well-differentiated.

The pooled estimates of the ORR are shown in Figure 2. The pooled ORR of the included studies was 15.5% (95% CI, 9.5–24.3%) (Figure 2A). Visual inspection of the funnel plot revealed no asymmetry when pooling the ORR across all studies (Figure S2), and no significant publication bias was evident according to Begg’s test (*p* = 0.79). Heterogeneity was present in pooling the ORR from all studies (I^2^ = 72%). When classified by primary tumor site (Figure 2B), the pooled ORRs were 9.5% (95% CI, 4.5–19.2%) in the GEP group and 24.7% (95% CI, 16.1–36.1%) in the thoracic group. The pooled ORRs of pancreatic NENs and gastrointestinal (GI) NENs were 11.4% (95% CI, 4.6–25.6%) and 9.0% (95% CI, 4.4–17.6%), respectively. Based on tumor differentiation (Figure 2C), the pooled ORR of the WD group was 10.4% (95% CI, 5.5–18.6%), while that of the PD group was higher (22.7%; 95% CI, 14.8–33.0%). The pooled ORR of the monotherapy group was 10.1% (95% CI, 5.0–19.3%), and that of the combination therapy group was 25.3% (95% CI, 17.1–35.7%) (Figure 2D).

Although the heterogeneity between studies decreased when pooling the ORR of each subgroup, it still existed in the GEP (I^2^ = 63%) and monotherapy group (I^2^ = 70%), which may be attributable to the presence of both PD and WD tumors in the same group. In addition, Klein’s study [36] reported an ORR of 40.0% for GEP tumors, far higher than any of the other studies (ranging from 4.8 to 15.6%). Patients included in Klein’s study were treated with combination ICI therapy (nivolumab and ipilimumab), whereas patients in the other studies were mostly treated with monotherapy. In the monotherapy group, studies by Lu [37] and Shirasawa [41] reported higher values for the ORR (20.0 and 38.5%, respectively) than the others (less than 9.8%). In Lu’s study, patients were eligible for inclusion if they had NEN with a high ki-67 index (≥10%). Shirasawa’s study included LCNEC of the lung only and was of the retrospective design. Marginal heterogeneity was noted in the WD group (I^2^ = 52%). There was no heterogeneity between the studies in the thoracic group, PD group, and the combination therapy group (I^2^ = 0%).

In the meta regression, multivariate analysis using the primary tumor site, tumor differentiation, and drug regimen as input variables revealed that all are significantly associated with the ORR. Thoracic tumors (OR, 2.6 (95% CI, 1.2–5.7)), PD tumors (OR, 2.6 [95% CI, 1.1–6.1]), and combination therapy (OR, 4.5 (95% CI, 1.9–10.3)) were associated with a higher ORR (Table 2).

**Figure 3:**
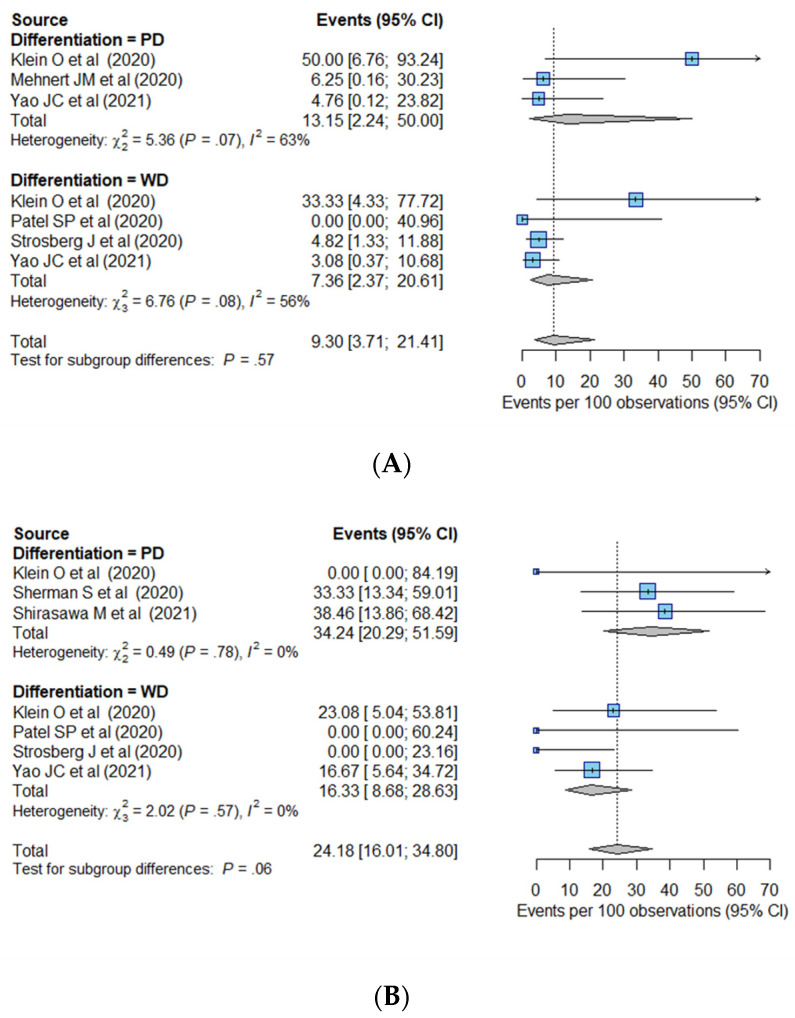
Sensitivity analysis of overall response rate according to tumor differentiation in the (**A**) GEP and (**B**) thoracic groups. CI, confidence interval; GEP, gastro–entero–pancreatic; PD, poorly differentiated; WD, well-differentiated.

**Figure 4:**
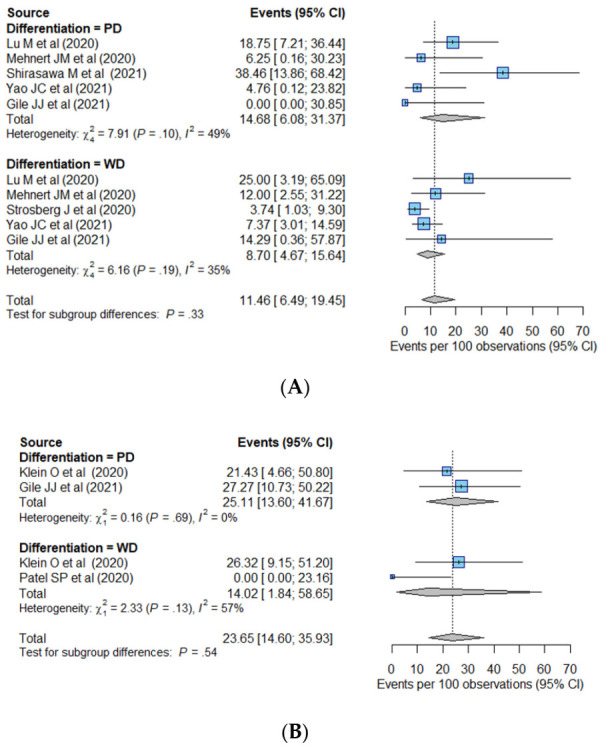
Sensitivity analysis according to tumor differentiation in the (**A**) monotherapy and (**B**) combination therapy groups. Abbreviations: CI, confidence interval; PD, poorly differentiated; WD, well-differentiated.

We performed sensitivity analyses to compare the pooled ORR values for the PD and WD tumors according to the primary tumor site (Figure 3) and drug regimen (Figure 4). In the GEP group, the PD tumors showed a pooled ORR of 13.2% (95% CI, 2.2–50.0%), which was 7.4% (95% CI, 2.4–20.6%) for the WD tumors. In the thoracic group, the pooled ORRs were 34.2% (95% CI, 20.3–51.6%) in the PD tumors and 16.3% (95% CI, 8.7–28.6%) in the WD tumors. According to the drug regimen, the pooled ORR was higher in PD tumors than in WD tumors in both the monotherapy group (14.7% (95% CI, 6.1–31.4%) in PD tumors and 8.7% (95% CI, 4.7–15.6%) in WD tumors) and combination therapy group (25.1% (95% CI, 13.6–41.7%) in PD tumors and 14.0% (95% CI, 1.8–58.7%) in WD tumors).

When separating WD tumors into grade 1–2 NETs and grade 3 NETs, the ORR of grade 1–2 NETs was extractable in five studies (which provided data of >5 patients with grade 1–2 NETs). The pooled ORR of grade 1–2 NETs was 7.7% (95% CI, 4.5–12.9%) (Figure S3), which was slightly lower than the pooled ORR of all WD NETs (10.4%). Two studies provided the response data of grade 3 NETs. In Klein’s study, two of three grade 3 NET patients achieved CR or PR (ORR, 66.7%) and in Gile’s study, none of three grade 3 NET patients achieved response (ORR, 0%).

#### 3.2.2. Survival-Related Endpoints

The IPD for the OS and PFS was reconstructed in eight of the included studies [19,36,38,39,40,41,42,43]. The IPD of 390 patients was reconstructed for the OS, and the median OS was 22.7 months (95% CI, 20.1–25.9 months). When divided by primary tumor site and tumor differentiation, the shortest median OS was noted for GEP PD tumors (6.8 months; 95% CI, 3.6–10.0 months), followed by GEP WD tumors (20.9 months; 95% CI, 20.1 months–not reached) and thoracic PD tumors (22.8 months; 95% CI, 12.3 months—not reached), and the longest median OS was noted for thoracic WD tumors (not reached) (Figure S4). The median OS of pancreatic NENs was 20.9 months (20.1 months—not reached) and was not reached for GI NENs. The median OSs of WD tumors and PD tumors were 23.7 months (95% CI, 19.3–28.0 months) and 12.3 months (95% CI, 8.0–23.8 months), respectively. With respect to the drug regimen, the data were not separable according to the primary tumor site or tumor differentiation variables. Stratified Cox regression analysis, which included the primary tumor site, tumor differentiation, and drug regimen as covariates, indicated that tumor differentiation was the only influencing factor for OS. PD tumors had a hazard ratio (HR) of 4.2 (95% CI, 2.0–8.7) (Table 3).

For the PFS, the IPD of 388 patients was reconstructed (median PFS, 3.8 months; 95% CI, 3.5–4.1 months). When divided by primary tumor site and tumor differentiation, GEP PD tumors showed the shortest median PFS (1.8 months; 95% CI, 1.7–2.0 months). GEP WD tumors and thoracic PD tumors showed median PFSs of 3.7 months (95% CI, 3.5–4.0 months) and 3.8 months (95% CI, 2.5–5.2 months), respectively, and the longest median PFS was noted for thoracic WD tumors (5.6 months; 95% CI, 4.6–6.6 months). The median PFS of pancreatic NENs was 3.8 months (3.2–4.3 months), and that of GI NENs was 3.5 months (1.7–5.3 months). The median PFSs of patients with WD tumors and PD tumors were 4.2 months (95% CI, 3.4–5.0 months) and 2.8 months (95% CI, 2.0–3.6 months), respectively. From stratified Cox-regression analysis, as found for the OS, tumor differentiation was significantly associated with the PFS, while primary tumor site and drug regimen were not. PD tumors had an HR of 2.6 (95% CI, 1.6–4.4) (Figure S5, Table 3).

## 4. Discussion

This study shows that, on average, ICIs show the ORR of 15.5% in patients with advanced or metastatic NENs. The primary tumor site, tumor differentiation, and drug regimen were found to affect the ORR. PD tumors showed a robustly higher response rate than WD tumors, although patients with PD tumors had poorer survival outcomes.

Given the large between-study heterogeneity in pooling the ORR from all 10 included studies, i.e., I^2^ of 72%, it would be not appropriate to merely accept the meta-analytic summary estimate of 15.5%. We explored the source of the heterogeneity by performing subgroup analyses and meta regression. The primary tumor site, tumor differentiation, and drug regimen significantly affected the ORR and were thus revealed as the causes of heterogeneity.

Our study results suggested that ICIs may show substantial antitumor activity against thoracic NENs. The pooled ORR was 24.7% for all thoracic NENs, 24.2% for WD thoracic tumors, and 34.2% for PD thoracic (all LCNEC) tumors. In this regard, Sabari et al. [44] recently reported that LCNEC of the lung treated with ICIs had an ORR of 26.7% (4/15), and a retrospective study from six French centers [45] stated that a PR of 60% (6/10) was achieved for ICI-treated advanced LCNEC of the lung, which also suggested considerable antitumor activity of ICI treatment in PD thoracic tumors as observed in our investigation. Previous studies have reported an ORR for first-line chemotherapy in carcinoid and LCNEC of the lung of 23 and 34–47% [46,47,48,49], respectively, and for second-line chemotherapy in LCNEC of the lung of 17% [50]. Although direct comparisons are limited as patients included in our study were more heavily treated with prior systemic therapies than the above-mentioned studies, we speculated that ICI treatments have the potential to demonstrate better antitumor activity in advanced thoracic NENs than conventional systemic treatments. To confirm this, randomized trials comparing ICIs to standard treatment options, including chemotherapeutics, are necessary.

The current classification of NENs differs between organ systems and causes considerable confusion [51]. To address this, a common framework for NEN classification was proposed by the International Agency for Research on Cancer (IARC) and WHO expert consensus meeting [52] which suggested distinction between differentiated NETs and NECs, irrespective of their site of origin. Hence, we separated grade 3 NETs from PD NECs and included them in the WD tumor group. In our analyses, a different response to ICI treatment was noted in accordance with tumor differentiation. The ORR of the PD group was 22.7%, compared to 10.4% in the WD group, and this trend of higher ORR of PD tumors was robust in terms of the primary tumor site and drug regimen. In other solid tumors, PD-L1 expression and TMB have been identified as potential biomarkers of the tumor response to immunotherapy [53,54]. In the case of NENs, few studies have reported that the expression of PD-1 and PD-L1 is higher in grade 3 NETs and NECs than in lower grade tumors [55] and that TMB was low in grade 1–2 NETs compared to that in NECs [56], indicating that the different response to ICI treatment in WD and PD tumors may be partly attributable to the different expression of immune checkpoints and TMB. We also tried to separate WD tumors into grade 1–2 NETs and grade 3 NETs to the extent available, which was limited as the number numbers of studies and patients were small. Further studies are necessary to clarify the relationship between these potential biomarkers and the response of NENs to ICI treatment.

A prior meta-analysis performed by Bongiovanni et al. [57] investigated the activity of ICIs in NENs; however, their results were inconsistent from ours. In their study, the pooled ORR was 10%, and there was no significant difference in ORR between WD NENs (11%) and PD NENs (12%). Thus, we infer that the main source of discrepancy was the different study eligibility criteria used. We excluded studies with a sample size of <10 patients and conference abstracts [58] and only included the single most recently published study among the studies with overlapped cohorts. The significantly higher pooled ORR in PD NENs than WD NENs, one of our main results, shares the same context with prior studies, which showed higher expression of PD-L1 and TMB in higher-grade NENs than in lower-grade NENs [53,54], which may explain the higher response rate in PD groups compared to WD groups.

Combination ICI regimens showed a higher ORR (25.3%, pooled) than the monotherapy (10.1%, pooled) in our meta-analyses. Three of our included studies evaluated the efficacy of nivolumab plus ipilimumab [35,36,39] and consistently observed a high ORR of 24.1–27.3%. There is now growing evidence that ICI monotherapy has limited effectiveness against NENs, particularly PD tumors [59,60,61], which is consistent with our observations. Only the nivolumab plus ipilimumab combination ICI regimen was evaluable in our study, however, and exploration of the synergistic effect of various combinations of ICIs versus other immunotherapeutic and different anticancer treatments is required.

In our study, the median OS and PFS of all IPD-retrieved patients were 22.7 and 3.8 months, respectively. In one prior phase 2 trial [62], the median OS of 18 NEN patients (14 WD and 4 PD; 22% previously treated) treated with irinotecan plus cisplatin was 11.4 months. In our study, thoracic PD tumors (LCNECs) showed a median OS of 22.8 months. In comparison, prior studies of thoracic LCNEC patients who received first-line chemotherapy have reported a median OS ranging from 8.0–12.6 months [48,49]. In our study, GEP PD tumors had a median OS of 6.8 months and a median PFS of 1.8 months, whereas one previous study reported that patients receiving second-line chemotherapy for an extrapulmonary PD NEN (mostly GEP origin) showed a median OS of 6.2 months and median PFS of 2.3 months [63]. The characteristics of the heavily treated advanced or metastatic NEN patients in our included studies made it difficult to compare the survival outcomes between our present investigation and others. Although it seems that ICI treatment has the potential to provide survival benefit in patients with NENs, especially PD tumors, randomized trials comparing ICIs and standard chemotherapies are necessary to clarify this issue.

Our study has some limitations. First, the number of included studies (10 in total) was small. However, all of the relevant original articles published to date were included in our meta-analysis. A total of 464 NEN patients were analyzed, and the individual participant survival data of more than 380 patients was reconstructed. Second, in terms of the primary tumor site, only those of thoracic or GEP origin were available for separate subgroup analysis. Other sites of origin were not evaluable, as the number of cases was too small and the required information was not fully presented, which is an inherent limitation of a systematic review. However, considering that the GEP and lung are the most frequent sites of NENs, comprising approximately 70 and 20% of all NENs, respectively [2], we believe that our analysis is clinically meaningful. Third, the included studies were all single-arm investigations that did not use a control group for comparison. Therefore, we could not address whether ICIs have better antitumor activity or provide a survival benefit compared to current standard therapies. However, our current results still provide useful insights on the role of ICIs in treatment of NEN and could serve as a basis for designing future randomized controlled trials. Fourth, the role of various combination regimens of ICI was not explored, as there were only three studies which included the combination regimen, and the same single combination regimen (ipilimumab plus nivolumab) was used in these studies. Further studies would be anticipated to clarify the role of ICI combination with various regimens in the treatment of NENs.

## 5. Conclusions

In conclusion, our current meta-analysis provides evidence-based measures of the treatment efficacy of ICIs in patients with advanced or metastatic NENs. The overall ORR was 15.5%, which was found to be influenced by the primary tumor site, tumor differentiation status, and by the drug regimen. PD tumors showed a robustly higher response rate than WD tumors but were associated with poorer survival outcomes.

## Figures and Tables

**Table 1:** Characteristics of the studies included in the meta-analysis.

Author (Year)	Design	Sample Size *	Primary Site	Grade/ Differentiation	Prior Systemic Therapy	Details of Prior Systemic Therapy	Disease State	ICI Regimen	Follow-Up ^†^ (Months)
Klein (2020)	Phase 2 Clinical Trial	29	Lung, thymus, GI, prostate, unknown	WD or PD (Low [*n* = 3], intermediate [*n* = 13], high [*n* = 13])	89.7%	Chemotherapy 86% (platinum/etoposide or temozolomide/capecitabine)	Advanced or metastatic	Ipilimumab + nivolumab	Up to 26
Lu (2020)	Phase 1b Clinical Trial	40	Pancreas, GI	WD (*n* = 8), PD (*n* = 32)	100%	PRRT 21%	Metastatic, Ki-67 ≥ 10%, PD on prior therapy	Toripalimab	Up to 24
Mehnert (2020)	Phase 1b Clinical Trial	41	Pancreas, lung, GI, others	WD	70.7%	Everolimus 7%	Advanced or metastatic, PD on prior therapy	Pembrolizumab	Up to 24
Patel (2020)	Phase 2 Clinical Trial	32	Lung, thymus, GI, cervix, prostate, unknown	WD or PD (low [*n* = 4], intermediate [*n* = 10], high [*n* = 18])	100%	Sunitinib 7%	PD on prior therapy and no available standard therapy	Ipilimumab + nivolumab	Up to 15
Sherman (2020)	Retrospective	18	Lung	Large cell NEC	NA	Pembrolizumab 3%	Advanced	Monotherapy (nivolumab, pembrolizumab, atezolizumab, or durvalumab) or Combination (nivolumab + Ipilimumab)	6.2
Shirasawa (2020)	Retrospective	13	Lung	Large cell NEC	NA	NA	Advanced or metastatic	Nivolumab or pembrolizumab	NA
Strosberg (2020)	Phase 2 Clinical Trial	107	Lung, pancreas, GI, liver, ovary, unknown	WD	97.2%	Chemotherapy 65.9%	Mostly metastatic (106/107), PD on prior therapy	Pembrolizumab	24.2
Vijayvergia (2020)	Phase 2 Clinical Trial	29	Thymus, pancreas, GI, kidney	Grade 3 WD or PD	100%	Everolimus 31.7%	Advanced or metastatic, PD on prior therapy	Pembrolizumab	Up to 36
Yao (2021)	Phase 2 Clinical Trial	116	Lung, thymus, pancreas, GI, gallbladder, unknown	WD (*n* = 95), PD (*n* = 21)	100%	Somatostatin analogues 29.3%	Metastatic, PD on prior therapy	Spartalizumab	13.4
Gile (2021)	Retrospective	39	Pancreas, GI, head and neck, bladder, uterus, gallbladder, unknown	WD (*n* = 7), PD (*n* = 32)	NA	Sunitinib 9.8%	Metastatic (81%)	Monotherapy (pembrolizumab, Nivolumab, or atezolizumab) or Combination (nivolumab + ipilimumab)	NEC, 10.7; NET, 79.8

* Number of patients treated with an ICI-based regimen whose tumor responses were evaluable according to RECIST 1.1; ^†^ Unless otherwise indicated, data are expressed as the median value; GI, gastrointestinal; ICI, immune checkpoint inhibitor; NA; not available; NEC, neuroendocrine carcinoma; NET, neuroendocrine tumor; PD, poorly differentiated; PRRT, peptide receptor radionuclide therapy; WD, well-differentiated.

**Table 2:** Meta-regression analysis of the overall response rate.

Variables	Pooled ORR (95% CI)	Univariate Analysis		Multivariate Analysis	
		OR (95% CI)	*p* Value	OR (95% CI)	*p* Value
**Overall (*n* = 426)**	15.5% (9.5–24.3%)	-		-	
**Primary site**			0.13		0.02
GEP (*n* = 266)	9.5% (4.5–19.2%)	Reference		Reference	
Thoracic (*n* = 97)	24.7% (16.1–36.1%) *	2.33 (0.75–7.22)	0.13	2.59 (1.18–5.72)	
**Differentiation**			0.08		0.03
WD (*n* = 291)	10.4% (5.5–18.6%)	Reference		Reference	
PD (*n* = 126)	22.7% (14.8–33.0%) *	2.72 (0.88–8.40)	0.08	2.60 (1.11–6.10)	
**Drug regimen**			0.002		0.001
Monotherapy (*n* = 363)	10.1% (5.0–19.3%)	Reference		Reference	
Combination (*n* = 83)	25.3% (17.1–35.7%) *	5.12 (1.97–13.61)	0.002	4.45 (1.92–10.33)	

* Significantly higher than the reference. CI, confidence interval; GEP, gastro–entero–pancreas; OR, odds ratio; PD, poorly differentiated; WD, well-differentiated.

**Table 3:** Stratified Cox regression analysis of overall survival and progression-free survival using individual patient data.

Survival Data	Variables	Univariate Analysis		Multivariate Analysis	
		HR (95% CI)	*p* Value	HR (95% CI)	*p* Value
Overall survival	**Primary site**		0.06		0.37
	Thoracic (*n* = 66)	Reference		Reference	
	GEP (*n* = 102)	2.27 (0.95–5.44)		1.53 (0.61–3.85)	
	**Differentiation**		<0.001		<0.001
	WD (*n* = 243)	Reference		Reference	
	PD (*n* = 57)	4.76 (2.40–9.44) *		4.20 (2.04–8.66)	
	**Drug regimen**		0.16		NA
	Monotherapy (*n* = 306)	Reference		Reference	
	Combination (*n* = 61)	1.37 (0.88–2.13)		NA	
Progression free survival	**Primary site**		0.02		0.27
	Thoracic (*n* = 86)	Reference		Reference	
	GEP (*n* = 127)	1.59 (1.07–2.35)		1.27 (0.83–1.95)	
	**Differentiation**		<0.001		<0.001
	WD (*n* = 276)	Reference		Reference	
	PD (*n* = 65)	3.00 (1.88–4.79) *		2.64 (1.59–4.37)	
	**Drug regimen**		0.51		NA
	Monotherapy (*n* = 306)	Reference		Reference	
	Combination (*n* = 61)	0.78 (0.38–1.61)		NA	

* Significantly higher than the reference. CI, confidence interval; GEP, gastro–entero–pancreas; HR, hazard ratio; NA, not available; PD, poorly differentiated; WD, well-differentiated.

## Data Availability

The datasets generated and/or analyzed during the current study are available from the corresponding author on reasonable request.

## Supplementary Materials

The following are available online at https://www.mdpi.com/article/10.3390/cancers14030794/s1: Table S1: Detailed search queries; Figure S1: Risk of bias summary of included studies; Figure S2: Funnel plots for visual assessment of publication bias; Figure S3: Pooled ORR of grade 1–2 NETs; Figure S4: Overall survival based on individual patient data; Figure S5: Progression-free survival based on individual patient data.

## References

[1] Yao J.C., Hassan M., Phan A., Dagohoy C., Leary C., Mares J.E., Abdalla E.K., Fleming J.B., Vauthey J.-N., Rashid A. (2008). One hundred years after “carcinoid”: Epidemiology of and prognostic factors for neuroendocrine tumors in 35,825 cases in the United States. J. Clin. Oncol..

[2] Maggio I., Manuzzi L., Lamberti G., Ricci A.D., Tober N., Campana D. (2020). Landscape and future perspectives of immunotherapy in neuroendocrine neoplasia. Cancers.

[3] Dasari A., Shen C., Halperin D., Zhao B., Zhou S., Xu Y., Shih T., Yao J.C. (2017). Trends in the incidence, prevalence, and survival outcomes in patients with neuroendocrine tumors in the United States. JAMA Oncol..

[4] Hallet J., Law C., Cukier M., Saskin R., Liu N., Singh S. (2014). Exploring the rising incidence of neuroendocrine tumors: A population-based analysis of epidemiology, metastatic presentation, and outcomes. Cancer.

[5] Chang J.S., Chen L.-T., Shan Y.-S., Chu P.-Y., Tsai C.-R., Tsai H.-J. (2021). An updated analysis of the epidemiologic trends of neuroendocrine tumors in Taiwan. Sci. Rep..

[6] Chauhan A., Kohn E., Del Rivero J. (2020). Neuroendocrine Tumors-Less Well Known, Often Misunderstood, and Rapidly Growing in Incidence. JAMA Oncol..

[7] National Comprehensive Cancer Network Neuroendocrine and Adrenal Tumors (Version 4. 2021). https://www.nccn.org/professionals/physician_gls/pdf/neuroendocrine.pdf.

[8] Yoo C., Oh C.R., Kim S.T., Bae W.K., Choi H.J., Oh D.Y., Lee M.A., Ryoo B.Y. (2021). Systemic Treatment of Advanced Gastroenteropancreatic Neuroendocrine Tumors in Korea: Literature Review and Expert Opinion. Cancer Res. Treat..

[9] Amin M.B., Edge S., Greene F., Byrd D.R., Brookland R.K., Washington M.K., Gershenwald J.E., Compton C.C., Hess K.R., Sullivan D.C. (2017). AJCC Cancer Staging Manual.

[10] Klimstra D., Klöppel G., La Rosa S., Rindi G. (2019). WHO Classification of Tumours: Digestive System Tumours.

[11] Chan D., Freixinos V.R., Doherty M., Wasson K., Iscoe N., Raskin W., Hallet J., Myrehaug S., Law C., Thawer A. (2021). Avelumab in Unresectable/Metastatic, Progressive, Grade 2–3 Neuroendocrine Neoplasms (NEN): Combined Results From NET-001 and NET-002 Trials. Pancreas.

[12] Sorbye H., Welin S., Langer S.W., Vestermark L.W., Holt N., Osterlund P., Dueland S., Hofsli E., Guren M., Ohrling K. (2013). Predictive and prognostic factors for treatment and survival in 305 patients with advanced gastrointestinal neuroendocrine carcinoma (WHO G3): The NORDIC NEC study. Ann. Oncol..

[13] Garcia-Carbonero R., Sorbye H., Baudin E., Raymond E., Wiedenmann B., Niederle B., Sedlackova E., Toumpanakis C., Anlauf M., Cwikla J. (2016). ENETS consensus guidelines for high-grade gastroenteropancreatic neuroendocrine tumors and neuroendocrine carcinomas. Neuroendocrinology.

[14] Vaddepally R.K., Kharel P., Pandey R., Garje R., Chandra A.B. (2020). Review of indications of FDA-approved immune checkpoint inhibitors per NCCN guidelines with the level of evidence. Cancers.

[15] Hellmann M.D., Ott P.A., Zugazagoitia J., Ready N.E., Hann C.L., De Braud F.G., Antonia S.J., Ascierto P.A., Moreno V., Atmaca A. (2017). Nivolumab (nivo) ± ipilimumab (ipi) in advanced small-cell lung cancer (SCLC): First report of a randomized expansion cohort from CheckMate 032. J. Clin. Oncol..

[16] Chung H.C., Lopez-Martin J.A., Kao S.C.-H., Miller W.H., Ros W., Gao B., Marabelle A., Gottfried M., Zer A., Delord J.-P. (2018). Phase 2 study of pembrolizumab in advanced small-cell lung cancer (SCLC): KEYNOTE-158. J. Clin. Oncol..

[17] Nghiem P., Bhatia S., Daud A., Friedlander P., Kluger H., Kohrt H., Kudchadkar R., Lipson E., Lundgren L., Margolin K. (2015). 22LBA Activity of PD-1 blockade with pembrolizumab as first systemic therapy in patients with advanced Merkel cell carcinoma. Eur. J. Cancer.

[18] Horn L., Mansfield A.S., Szczęsna A., Havel L., Krzakowski M., Hochmair M.J., Huemer F., Losonczy G., Johnson M.L., Nishio M. (2018). First-line atezolizumab plus chemotherapy in extensive-stage small-cell lung cancer. N. Engl. J. Med..

[19] Vijayvergia N., Dasari A., Deng M., Litwin S., Al-Toubah T., Alpaugh R.K., Dotan E., Hall M.J., Ross N.M., Runyen M.M. (2020). Pembrolizumab monotherapy in patients with previously treated metastatic high-grade neuroendocrine neoplasms: Joint analysis of two prospective, non-randomised trials. Br. J. Cancer.

[20] Moher D., Liberati A., Tetzlaff J., Altman D.G. (2010). Preferred reporting items for systematic reviews and meta-analyses: The PRISMA statement. Int. J. Surg..

[21] Stewart L.A., Clarke M., Rovers M., Riley R.D., Simmonds M., Stewart G., Tierney J.F. (2015). Preferred reporting items for a systematic review and meta-analysis of individual participant data: The PRISMA-IPD statement. JAMA.

[22] Schardt C., Adams M.B., Owens T., Keitz S., Fontelo P. (2007). Utilization of the PICO framework to improve searching PubMed for clinical questions. BMC Med. Inf. Decis. Mak..

[23] Eisenhauer E.A., Therasse P., Bogaerts J., Schwartz L.H., Sargent D., Ford R., Dancey J., Arbuck S., Gwyther S., Mooney M. (2009). New response evaluation criteria in solid tumours: Revised RECIST guideline (version 1.1). Eur. J. Cancer.

[24] Ulbright T., Amin M., Balzer B., Berney D., Epstein J., Guo C., Idrees M., Looijenga L., Paner G., Rajpert-De Meyts E. (2016). WHO Classification of Tumours of the Urinary System and Male Genital Organs.

[25] Gilks C. (2014). World Health Organization Classification of Tumours: Pathology and Genetics of Tumours of Female Genital Organs.

[26] Travis W.D., Brambilla E., Burke A.P., Marx A., Nicholson A.G. (2015). WHO Classification of Tumours of the Lung, Pleura, Thymus and Heart.

[27] Villaruz L.C., Socinski M.A. (2013). The clinical viewpoint: Definitions, limitations of RECIST, practical considerations of measurement. Clin. Cancer Res..

[28] Barat M., Nguyen T.T.L., Hollande C., Coty J.B., Hoeffel C., Terris B., Dohan A., Mallet V., Pol S., Soyer P. (2021). LI-RADS v2018 major criteria: Do hepatocellular carcinomas in non-alcoholic steatohepatitis differ from those in virus-induced chronic liver disease on MRI?. Eur. J. Radiol..

[29] Guyot P., Ades A., Ouwens M.J., Welton N.J. (2012). Enhanced secondary analysis of survival data: Reconstructing the data from published Kaplan-Meier survival curves. BMC Med. Res. Methodol.

[30] Sterne J.A., Hernan M.A., Reeves B.C., Savovi J., Berkman N.D., Viswanathan M., Henry D., Altman D.G., Ansari M.T., Boutron I. (2016). ROBINS-I: A tool for assessing risk of bias in non-randomised studies of interventions. BMJ.

[31] DerSimonian R., Laird N. (1986). Meta-analysis in clinical trials Control Clin Trials. Control. Clin. Trials.

[32] Higgins J.P., Thompson S.G., Deeks J.J., Altman D.G. (2003). Measuring inconsistency in meta-analyses. BMJ.

[33] Vandenbroucke J.P. (1998). Bias in meta-analysis detected by a simple, graphical test. Experts’ views are still needed. BMJ.

[34] Riley R.D., Lambert P.C., Abo-Zaid G. (2010). Meta-analysis of individual participant data: Rationale, conduct, and reporting. BMJ.

[35] Gile J.J., Liu A.J., McGarrah P.W., Eiring R.A., Hobday T.J., Starr J.S., Sonbol M.B., Halfdanarson T.R. (2021). Efficacy of Checkpoint Inhibitors in Neuroendocrine Neoplasms: Mayo Clinic Experience. Pancreas.

[36] Klein O., Kee D., Markman B., Michael M., Underhill C., Carlino M.S., Jackett L., Lum C., Scott C., Nagrial A. (2020). Immunotherapy of ipilimumab and nivolumab in patients with advanced neuroendocrine tumors: A subgroup analysis of the CA209-538 clinical trial for rare cancers. Clin. Cancer Res..

[37] Lu M., Zhang P., Zhang Y., Li Z., Gong J., Li J., Li J., Li Y., Zhang X., Lu Z. (2020). Efficacy, safety, and biomarkers of Toripalimab in patients with recurrent or metastatic neuroendocrine neoplasms: A Multiple-Center phase Ib trial. Clin. Cancer Res..

[38] Mehnert J.M., Bergsland E., O’Neil B.H., Santoro A., Schellens J.H., Cohen R.B., Doi T., Ott P.A., Pishvaian M.J., Puzanov I. (2020). Pembrolizumab for the treatment of programmed death-ligand 1-positive advanced carcinoid or pancreatic neuroendocrine tumors: Results from the KEYNOTE-028 study. Cancer.

[39] Patel S.P., Othus M., Chae Y.K., Giles F.J., Hansel D.E., Singh P.P., Fontaine A., Shah M.H., Kasi A., Al Baghdadi T. (2020). A phase II basket trial of dual anti-CTLA-4 and anti-PD-1 blockade in rare tumors (DART SWOG 1609) in patients with nonpancreatic neuroendocrine tumors. Clin. Cancer Res..

[40] Sherman S., Rotem O., Shochat T., Zer A., Moore A., Dudnik E. (2020). Efficacy of immune check-point inhibitors (ICPi) in large cell neuroendocrine tumors of lung (LCNEC). Lung Cancer.

[41] Shirasawa M., Yoshida T., Takayanagi D., Shiraishi K., Yagishita S., Sekine K., Kanda S., Matsumoto Y., Masuda K., Shinno Y. (2021). Activity and Immune Correlates of Programmed Death-1 Blockade Therapy in Patients with Advanced Large Cell Neuroendocrine Carcinoma. Clin. Lung Cancer.

[42] Strosberg J., Mizuno N., Doi T., Grande E., Delord J.-P., Shapira-Frommer R., Bergsland E., Shah M., Fakih M., Takahashi S. (2020). Efficacy and safety of pembrolizumab in previously treated advanced neuroendocrine tumors: Results from the phase II KEYNOTE-158 study. Clin. Cancer Res..

[43] Yao J.C., Strosberg J., Fazio N., Pavel M.E., Bergsland E., Ruszniewski P., Halperin D.M., Li D., Tafuto S., Raj N. (2021). Spartalizumab in metastatic, well/poorly differentiated neuroendocrine neoplasms. Endocr. Relat. Cancer.

[44] Sabari J.K., Julian R.A., Ni A., Halpenny D., Hellmann M.D., Drilon A.E., Li B.T., Poirier J.T., Rudin C.M., Rekhtman N. (2018). Outcomes of advanced pulmonary large cell neuroendocrine carcinoma stratified by RB1 loss, SLFN11 expression, and tumor mutational burden. J. Clin. Oncol..

[45] Levra M.G., Mazieres J., Valette C.A., Molinier O., Planchard D., Frappat V., Ferrer L., Toffart A.C., Moro-Sibilot D. (2017). P1. 07-012 efficacy of immune checkpoint inhibitors in large cell neuroendocrine lung cancer: Results from a French retrospective cohort: Topic: Drug treatment alone and in combination with radiotherapy. J. Thorac. Oncol..

[46] Forde P.M., Hooker C.M., Boikos S.A., Petrini I., Giaccone G., Rudin C.M., Yang S.C., Illei P.B., Hann C.L., Ettinger D.S. (2014). Systemic therapy, clinical outcomes, and overall survival in locally advanced or metastatic pulmonary carcinoid: A brief report. J. Thorac. Oncol..

[47] Chong C.R., Wirth L.J., Nishino M., Chen A.B., Sholl L.M., Kulke M.H., McNamee C.J., Jänne P.A., Johnson B.E. (2014). Chemotherapy for locally advanced and metastatic pulmonary carcinoid tumors. Lung Cancer.

[48] Niho S., Kenmotsu H., Sekine I., Ishii G., Ishikawa Y., Noguchi M., Oshita F., Watanabe S.-i., Nakajima R., Tada H. (2013). Combination chemotherapy with irinotecan and cisplatin for large-cell neuroendocrine carcinoma of the lung: A multicenter phase II study. J. Thorac. Oncol..

[49] Le Treut J., Sault M., Lena H., Souquet P., Vergnenegre A., Le Caer H., Berard H., Boffa S., Monnet I., Damotte D. (2013). Multicentre phase II study of cisplatin-etoposide chemotherapy for advanced large-cell neuroendocrine lung carcinoma: The GFPC 0302 study. Ann. Oncol..

[50] Shimada Y., Niho S., Ishii G., Hishida T., Yoshida J., Nishimura M., Yoh K., Goto K., Ohmatsu H., Ohe Y. (2012). Clinical features of unresectable high-grade lung neuroendocrine carcinoma diagnosed using biopsy specimens. Lung Cancer.

[51] La Rosa S., Uccella S. (2020). Classification of neuroendocrine neoplasms: Lights and shadows. Rev. Endocr. Metab. Disord..

[52] Rindi G., Klimstra D.S., Abedi-Ardekani B., Asa S.L., Bosman F.T., Brambilla E., Busam K.J., de Krijger R.R., Dietel M., El-Naggar A.K. (2018). A common classification framework for neuroendocrine neoplasms: An International Agency for Research on Cancer (IARC) and World Health Organization (WHO) expert consensus proposal. Mod. Pathol..

[53] Rizvi H., Sanchez-Vega F., La K., Chatila W., Jonsson P., Halpenny D., Plodkowski A., Long N., Sauter J.L., Rekhtman N. (2018). Molecular determinants of response to anti-programmed cell death (PD)-1 and anti-programmed death-ligand 1 (PD-L1) blockade in patients with non-small-cell lung cancer profiled with targeted next-generation sequencing. J. Clin. Oncol..

[54] Samstein R.M., Lee C.-H., Shoushtari A.N., Hellmann M.D., Shen R., Janjigian Y.Y., Barron D.A., Zehir A., Jordan E.J., Omuro A. (2019). Tumor mutational load predicts survival after immunotherapy across multiple cancer types. Nat. Genet..

[55] Kim S.T., Ha S.Y., Lee S., Ahn S., Lee J., Park S.H., Park J.O., Lim H.Y., Kang W.K., Kim K.-M. (2016). The impact of PD-L1 expression in patients with metastatic GEP-NETs. J. Cancer.

[56] Cives M., Quaresmini D., Rizzo F.M., Tucci M., Silvestris F. (2019). The tumor microenvironment in neuroendocrine tumors: Biology and therapeutic implications. Neuroendocrinology.

[57] Bongiovanni A., Maiorano B.A., Azzali I., Liverani C., Bocchini M., Fausti V., Di Menna G., Grassi I., Sansovini M., Riva N. (2021). Activity and Safety of Immune Checkpoint Inhibitors in Neuroendocrine Neoplasms: A Systematic Review and Meta-Analysis. Pharmaceuticals.

[58] Scherer R.W., Saldanha I.J. (2019). How should systematic reviewers handle conference abstracts? A view from the trenches. Syst. Rev..

[59] Chan J.A., Raj N.P., Aggarwal R.R., Calabrese S., DeMore A., Dhawan M.S., Fattah D., Fong L., Grabowsky J., Hope T.A. (2021). Phase II study of pembrolizumab-based therapy in previously treated extrapulmonary poorly differentiated neuroendocrine carcinomas: Results of Part B (pembrolizumab + chemotherapy). J. Clin. Oncol..

[60] Fang L., Arvind D., Dowlati A., Mohamed A. (2021). Role of immunotherapy in gastro-enteropancreatic neuroendocrine neoplasms (gep-nens): Current advances and future directions. J. Neuroendocr..

[61] Mulvey C., Raj N.P., Chan J.A., Aggarwal R.R., Cinar P., Hope T.A., Kolli K., Zhang L., Calabrese S., Grabowsky J.A. (2019). Phase II study of pembrolizumab-based therapy in previously treated extrapulmonary poorly differentiated neuroendocrine carcinomas: Results of Part A (pembrolizumab alone). J. Clin. Oncol..

[62] Kulke M.H., Wu B., Ryan D.P., Enzinger P.C., Zhu A.X., Clark J.W., Earle C.C., Michelini A., Fuchs C.S. (2006). A phase II trial of irinotecan and cisplatin in patients with metastatic neuroendocrine tumors. Dig. Dis. Sci..

[63] McGarrah P.W., Leventakos K., Hobday T.J., Molina J.R., Finnes H.D., Westin G.F., Halfdanarson T.R. (2020). Efficacy of second-line chemotherapy in extrapulmonary neuroendocrine carcinoma. Pancreas.

