# Supplementary material for: Efficacy of Immune Checkpoint Inhibitors against Advanced or Metastatic Neuroendocrine Neoplasms: A Systematic Review and Meta-Analysis
Source: Cancers (Basel). 2022 Feb 4;14(3):794. doi: 10.3390/cancers14030794 (PMC8833825; doi:10.3390/cancers14030794)
Supplement: Supplementary file 1 [file cancers-14-00794-s001.zip › cancers-1538227-supplementary.pdf]

Table S1. Detailed search queries

No.	Search queries for MEDLINE
#1	neuroendocrine[Title/Abstract] OR carcinoid[Title/Abstract] OR NET[Title/Abstract] OR NEC[Title/Abstract]
#2	immunother*[Title/Abstract] OR "immune checkpoint"[Title/Abstract] OR "checkpoint"[Title/Abstract] OR "PD-1"[Title/Abstract] OR "PD-L1"[Title/Abstract] OR "CTLA-4"[Title/Abstract] OR nivolumab[Title/Abstract] OR pembrolizumab[Title/Abstract] OR Spartalizumab[Title/Abstract] OR cemiplimab[Title/Abstract] OR Sintilimab[Title/Abstract] OR Tislelizumab[Title/Abstract] OR Toripalimab[Title/Abstract] OR Dostarlimab[Title/Abstract] OR AMP-224[Title/Abstract] OR atezolizumab[Title/Abstract] OR avelumab[Title/Abstract] OR durvalumab[Title/Abstract] OR KN035[Title/Abstract] OR AUNP12[Title/Abstract] OR CA-170[Title/Abstract] OR ipilimumab[Title/Abstract]
#3	#1 AND #2
#4	("2009/01/01"[Date - Publication] : "2021/04/01"[Date - Publication]) AND #3
No.	Search queries for EMBASE
#1	neuroendocrine:ab,ti OR carcinoid:ab,ti OR net:ab,ti OR nec:ab,ti
#2	immunotherapy:ab,ti OR checkpoint:ab,ti OR pd1:ab,ti OR 'pd 1':ab,ti OR ctla4:ab,ti OR 'ctla 4':ab,ti OR nivolumab:ab,ti OR pembrolizumab:ab,ti OR spartalizumab:ab,ti OR cemiplimab:ab,ti OR sintilimab:ab,ti OR tislelizumab:ab,ti OR toripalimab:ab,ti OR dostarlimab:ab,ti OR 'amp 224':ab,ti OR atezolizumab:ab,ti OR avelumab:ab,ti OR durvalumab:ab,ti OR kn035:ab,ti OR aunp12:ab,ti OR 'ca 170':ab,ti OR ipilimumab:ab,ti
#3	#1 AND #2
#4	#3 AND [2009-2021]/py AND [english]/lim

Figure S1. Risk of bias summary of included studies

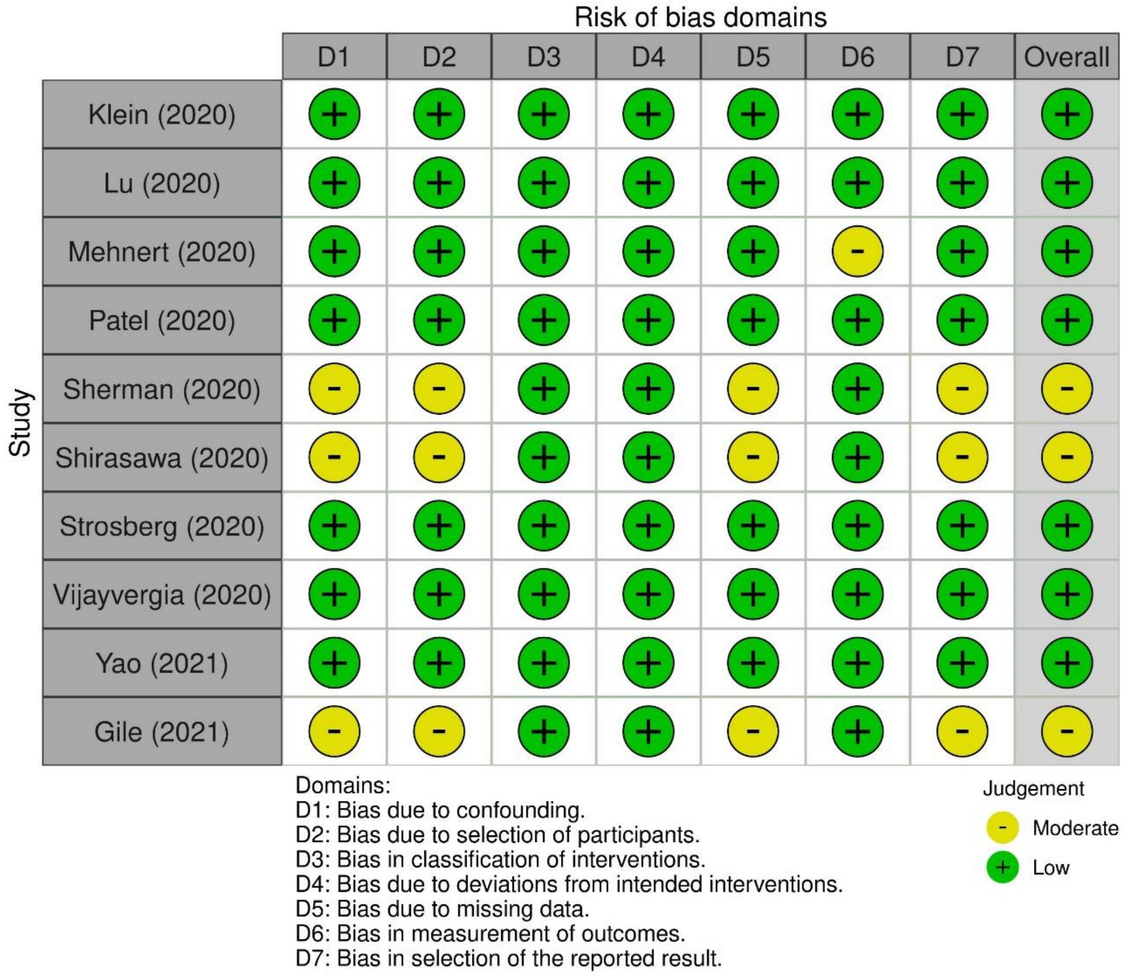

Figure S2. Funnel plots for visual assessment of publication bias

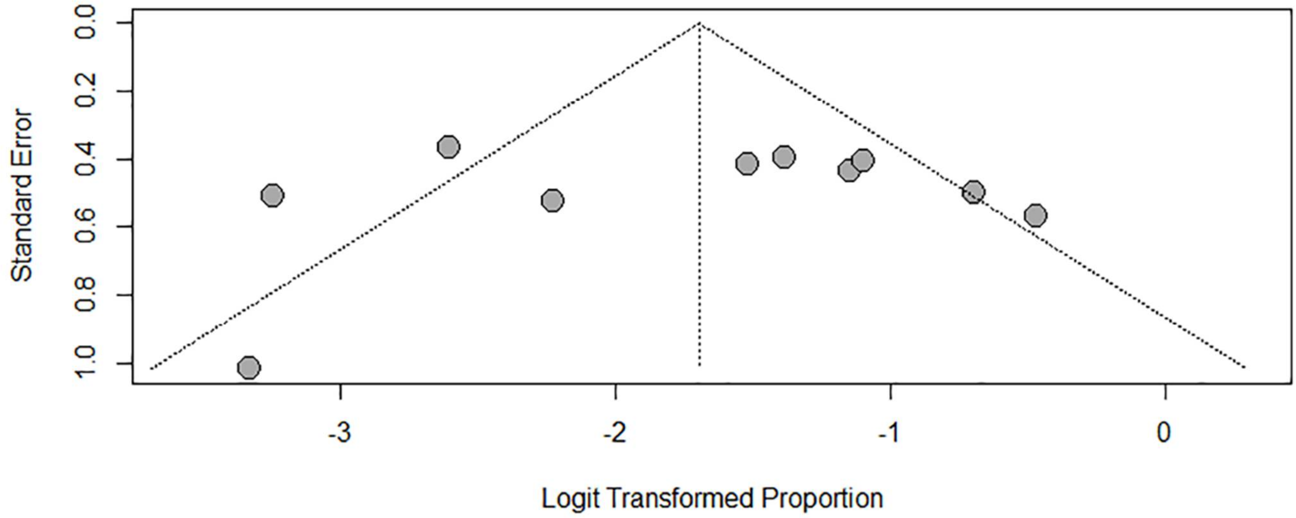

Figure S3. Pooled ORR of grade 1-2 NETs.

Source	Events (95% CI)
Klein O et al	18.75 [4.05; 45.65]
Mehnert JM et al	9.76 [2.72; 23.13]
Patel SP et al	0.00 [0.00; 28.49]
Strosberg J et al	3.74 [1.03; 9.30]
Yao JC et al	7.37 [3.01; 14.59]
Total	7.74 [4.53; 12.93]

Heterogeneity: $\chi^2_4 = 5.23$ (P = .26), I^2 = 24%

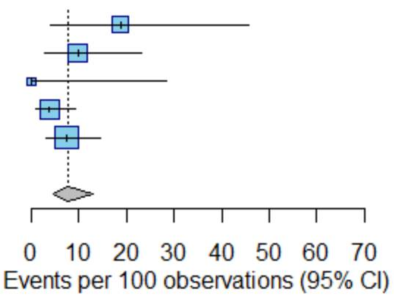

The pooled ORR of grade 1-2 NETs was 7.7% (95% CI, 4.5–12.9%).
CI, confidence interval; NET, neuroendocrine tumor; ORR, overall response rate

Figure S4. Overall survival based on individual patient data.

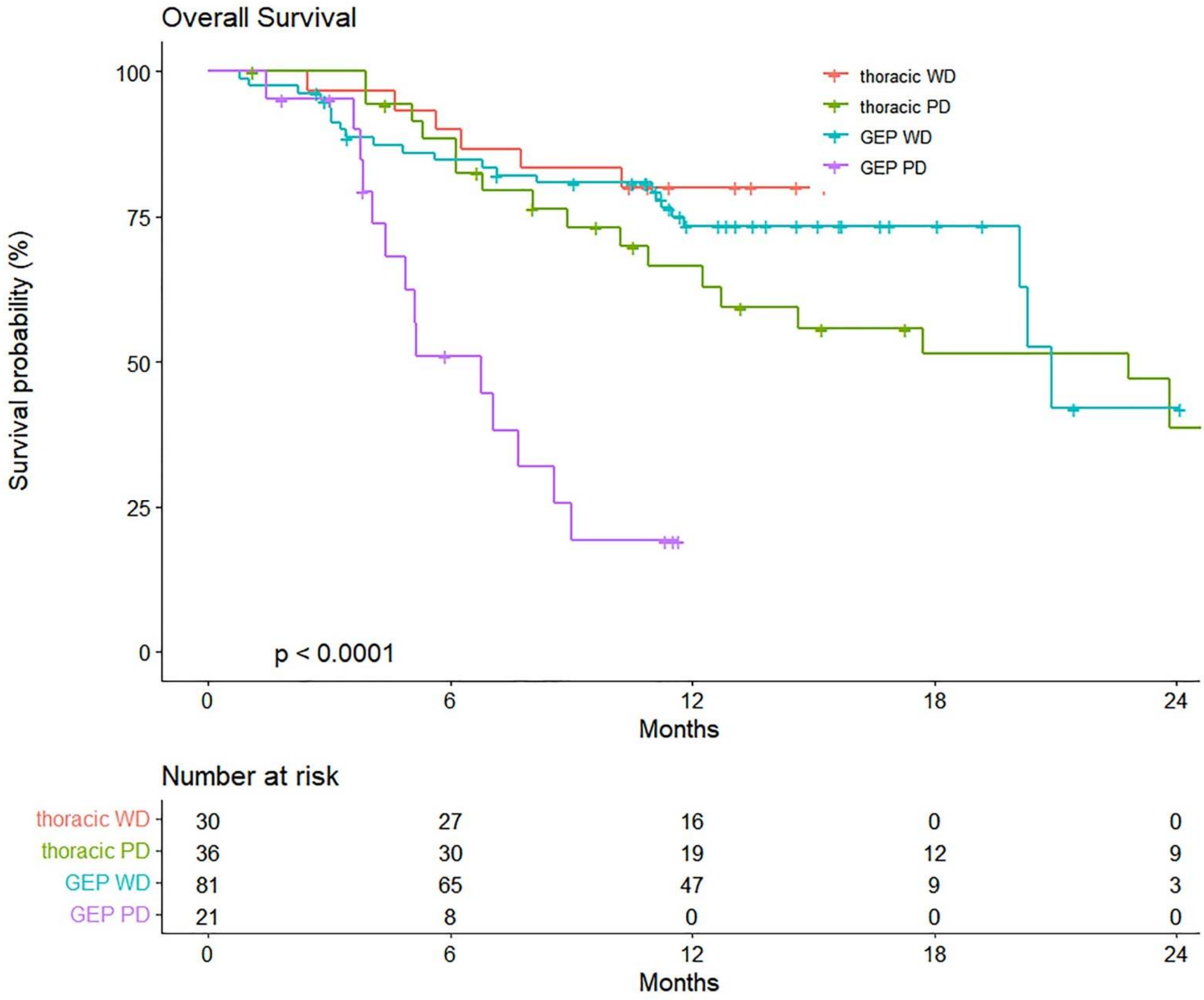

The median OS was 6.8 months (95% CI, 3.6–10 months) for the GEP PD tumors, 22.8 months (95% CI, 12.3 months–not reached) for the thoracic PD tumors, 20.9 months (95% CI, 20.1 months–not reached) for the GEP WD tumors, and not reached in thoracic WD tumors.

Abbreviations: CI, confidence interval; GEP, gatroenteropancreatic; OS, overall survival; PD, poorly differentiated; WD, well differentiated

Figure S5. Progression-free survival based on individual patient data.

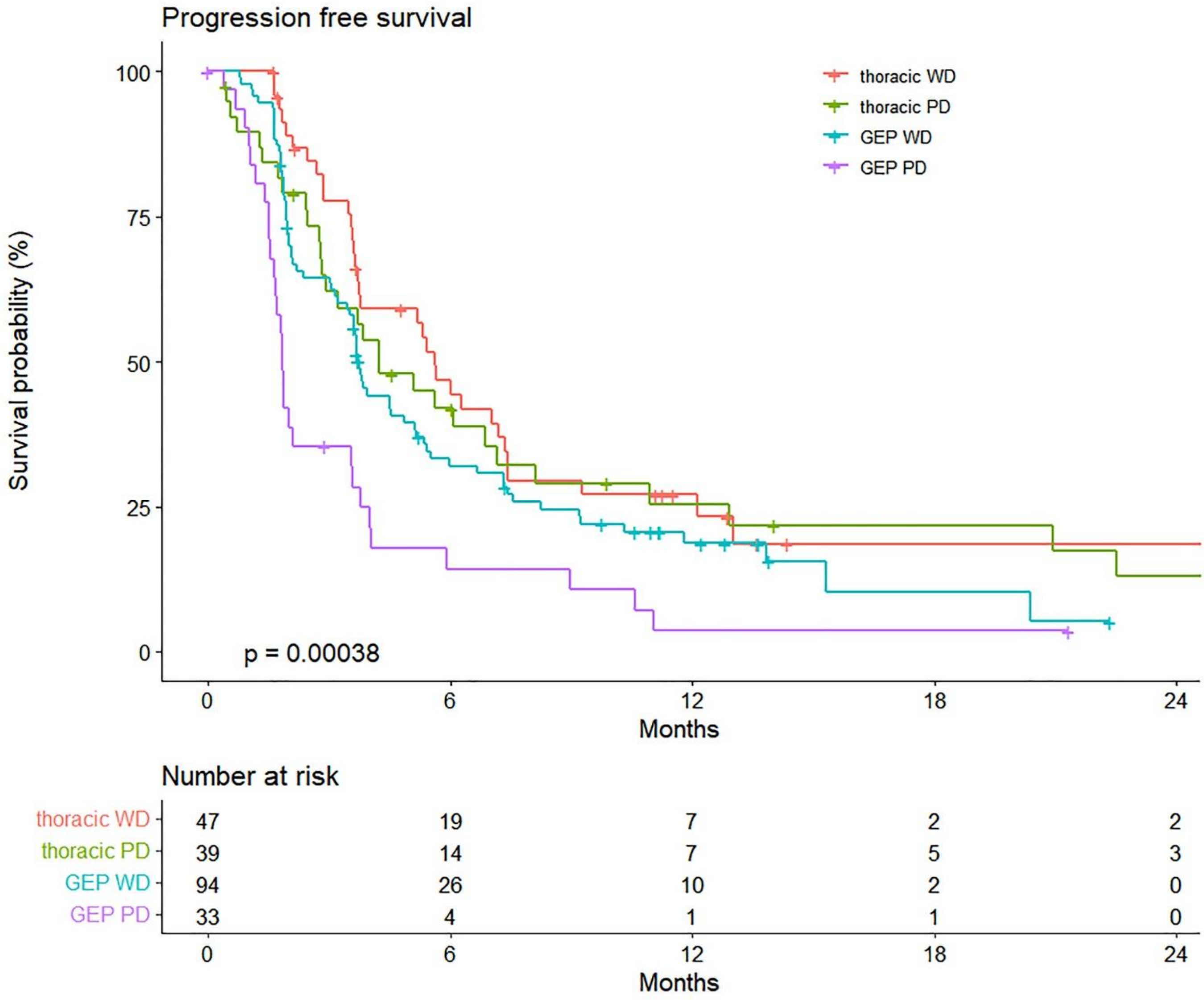

GEP PD tumors showed the shortest median PFS (1.8 months; 95% CI, 1.7–2.0 months), followed by GEP WD tumors (3.7 months; 95% CI, 3.5–4.0 months), and thoracic PD tumors(3.8 months; 95% CI, 2.5–5.2 months). The longest median PFS was noted in thoracic WD tumors (5.6 months; 95% CI, 4.6–6.6 months).

Abbreviations: CI, confidence interval; GEP, gatroenteropancreatic; PD, poorly differentiated; PFS, progression free survival; WD, well differentiated
